# Defining Exposure Predictors of Meropenem That Are Associated with Improved Survival for Severe Bacterial Infection: A Preclinical PK/PD Study in Sepsis Rat Model

**DOI:** 10.3390/antibiotics11111660

**Published:** 2022-11-19

**Authors:** Yan Wang, Lanyu Liu, Qiping Wu, Qiufen Yin, Feifan Xie

**Affiliations:** Xiangya School of Pharmaceutical Sciences, Central South University, Tongzipo Road 172, Changsha 410013, China

**Keywords:** pharmacokinetics, PK/PD, survival, meropenem, sepsis rat model

## Abstract

**Background**: The pharmacokinetic/pharmacodynamic (PK/PD) index of carbapenems that best correlates with in vivo antimicrobial activity is percent time of dosing interval in which free drug concentration remains above MIC (%*f*T > MIC), while the magnitudes of the PK/PD index of carbapenems remains undefined in critically ill sepsis patients. **Methods**: A sepsis rat model was first developed by comparing the survival outcomes after intraperitoneal injection of different inoculum size (1–10 × 10^7^ CFU) of *Pseudomonas aeruginosa* ATCC9027 (MIC = 0.125 mg/L) in neutropenic rats. The PK characteristics of the model drug meropenem in the developed sepsis rat model was then evaluated, and PK modeling and simulation was applied to design meropenem dosing regimens attaining various PD targets (40%*f*T > MIC, 100%*f*T > MIC, and 100%*f*T > 4 × MIC). The microbiological response and survival outcomes for different meropenem treatment regimens were investigated in the rat sepsis model (*n* = 12 for each group). **Results**: The optimal inoculum for the rat sepsis model was 1 × 10^7^ CFU of *Pseudomonas aeruginosa* ATCC9027. A one-compartment model with first-order absorption best described the PK of meropenem in sepsis rats. Pronounced survival prolongation and lower hazard risk were observed in the treatment groups of 50 or 75 mg/kg/q2.4h (100%*f*T > MIC) and 75 mg/kg/q2h (100%*f*T > 4 × MIC) compared to the 75 mg/kg/q6h (40%*f*T > MIC) group, while meropenem groups with PD targets of 100%*f*T > MIC and 100%*f*T > 4 × MIC showed comparable survival curves. Microbiological response for different PD targets is inconclusive due to irregular bacterial counts in blood samples. **Conclusions**: The PD target of 40%*f*T > MIC is suboptimal for sepsis rats, and the aggressive 100%*f*T > 4 × MIC target does not provide a survival benefit against the target of 100%*f*T > MIC.

## 1. Introduction

Sepsis is defined as a life-threatening organ dysfunction caused by a dysregulated host response to infection [1], and it remains a high mortality and morbidity among patients in intensive care units (ICUs) [2]. There are more than 30 million sepsis patients worldwide every year, leading to about 6 million deaths, with increasing incidence year by year [3]. Sepsis poses a serious threat to public health, as well as a huge economic burden to patients and medical organizations [4].

From the point of pathophysiological progress, sepsis is considered to evolve from an initial proinflammatory burst that leads to a cytokine storm, followed by a compensatory immunosuppressed response, both of which are responsible for increased mortality [5]. Naturally, sepsis is triggered by infection factors which have progressed to an uncontrolled immune response. Therefore, it is critical to suppress infection progress for sepsis treatment. The infection source of sepsis includes bacteria, virus, fungi, and others, with bacteria predominating [6]. Early and appropriate antibiotics administration is one of most effective interventions for the therapy and prognosis of patients with sepsis [7]. A number of studies have shown that initiating antibiotics as early as possible is highly associated with lower risk of death in severe sepsis patients [8,9,10]. Each hour of delay before administration of antimicrobials is associated with about 1% increased odds of mortality with severe sepsis [10]. There is a general consensus that the pharmacokinetics in sepsis patients is quite different from that in healthy populations. For example, sepsis involves the increased volume of distribution for drugs due to the vasodilatation caused by cytokine burst [5]. Therefore, careful and adequate antibiotic dosing is also important for improving sepsis patients’ outcome. Inadequate antibiotic exposure could fail to provide desired bacteria killing and often lead to the emergence of resistance. Currently, the pharmaceutical industry has little incentive to develop new or advanced antimicrobial agents for resistant infections due to the low returns on investment [11]. As a result, optimizing the dosing regimen of existing antibiotics remains a supplemental strategy to ensure that the regimen selected is effective while minimizing the risk of toxicity and development of drug resistance [12].

Bacterial sepsis patients often present with polymicrobial infections with both aerobic and anaerobic pathogens. The empirical therapy often includes gram-negative with additional anaerobic coverage, using a beta-lactam/beta-lactamase inhibitor combination or, a more advanced option, a carbapenem [7]. Carbapenems are notable for their broad spectrum of activity and their ability to inhibit beta-lactamase enzymes [13]. Imipenem and meropenem are the two most commonly used carbapenems in clinical practice, and meropenem is reported with less seizure proclivity.

As time-dependent antibiotics, the pharmacokinetic/pharmacodynamic (PK/PD) index of carbapenems that best correlates with their in vivo antimicrobial activity is the percent time of dosing interval in which free drug concentration remains above MIC (%*f*T > MIC) [14]. Currently, the magnitude of PK/PD index (i.e., PK/PD target) of carbapenems in critically ill sepsis patients is not clearly defined. The 40%*f*T > MIC target of carbapenems derived from the neutropenic thigh infection model is commonly used in patients with mild or moderate infection [15]. The utility of the 40%*f*T > MIC target was questioned in critically ill patients, as this population usually has an immunocompromised condition and severe infections. Accordingly, more aggressive PK/PD targets, such as 100%*f*T > MIC or even 100%*f*T > 4-6 × MIC, were proposed for critically ill patients [16,17,18,19]. These two targets were shown to provide better a clinical cure and/or bacteriological eradication in patients with serious bacterial infections. However, these retrospective clinical analyses were often based on a small number of events (i.e., failures at specific exposures) and non-actual patient concentration data [16,17]. The stringent PK/PD target of 100%*f*T > 4 × MIC also has the ability to prevent the development of resistance based on in vitro pharmacodynamics research [18].

It is difficult to determine the optimal PK/PD target of carbapenems for critically ill sepsis patients in clinical practice because of the complication of designing variables for controlled clinical trials for pharmacodynamic analyses. In this study, we intended to perform a systemic PK/PD analysis for the microbiological and survival outcomes of different PK/PD targets (40%*f*T > MIC, 100%*f*T > MIC, and 100%*f*T > 4 × MIC) in a sepsis rat model using meropenem as the model drug.

## 2. Results

### 2.1. In Vitro Susceptibility Testing

The determined MIC of meropenem for *Pseudomonas aeruginosa* ATCC 9027 was 0.125 μg/mL, and the MIC of *Pseudomonas aeruginosa* ATCC 27853 was 0.5 μg/mL, complying with the quality control criteria of CLSI.

### 2.2. Development of Sepsis Rat Model

**Figure 1:**
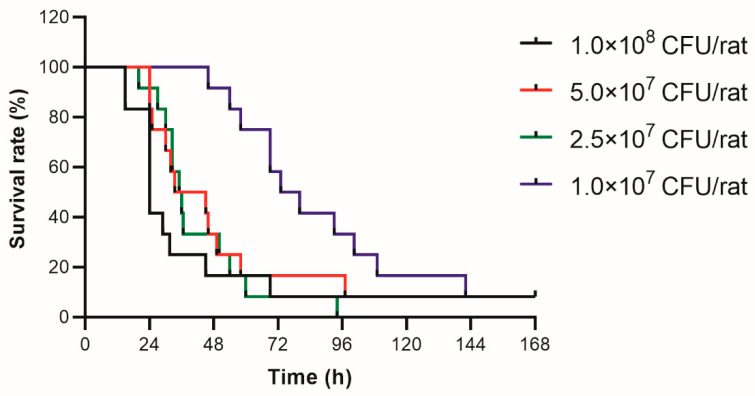
Survival curves of neutropenic rats 7 days after infection by intraperitoneal injection of one milliliter of different concentrations (1 × 10^8^ CFU/mL, 5 × 10^7^ CFU/mL, 2.5 × 10^7^ CFU/mL, and 1 × 10^7^ CFU/mL) of *Pseudomonas aeruginosa* ATCC9027.

The survival curves for the treatment and control groups are shown in Figure 1. The median survival times of experimental groups infected with 1 × 10^8^ CFU, 5 × 10^7^ CFU, 2.5 × 10^7^ CFU, and 1 × 10^7^ CFU of *Pseudomonas aeruginosa* ATCC 9027 were 24, 39.25, 35.5, and 76.5 h, respectively. As for the 3-day survival rate, the animal group infected with 1 × 10^7^ CFU was about 50%, while the remaining treatment groups were below 20%. As expected, the control animals all survived. Considering these results, the inoculum size of 1 × 10^7^ CFU of the organism was used for developing the sepsis rat model.

### 2.3. Pharmacokinetic Parameters of Meropenem and Dosing Regimen Determination

A one-compartment model with first-order absorption was optimal for the dataset. The mean values of apparent volume of distribution (V_d_), absorption rate constant (K_a_), and elimination rate constant (K_e_) of meropenem in sepsis rat were 0.326 L, 3.602 h^−1^, and 3.212 h^−1^, respectively.

**Figure 2:**
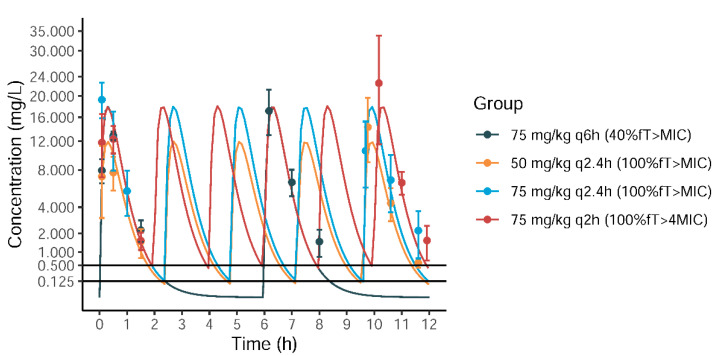
The simulated concentration–time curves and illustrative PD target attainment for different meropenem dosing regimens of 75 mg/kg/q6h (40%*f*T > MIC), 50 mg/kg/q2.4h (100%*f*T > MIC), 75 mg/kg/q2.4h (100%*f*T > MIC), and 75 mg/kg/q2h (100%*f*T > 4 × MIC) for a treatment duration of 12 h. The dots represent the observed mean ± SD concentrations, and the Y-axis is shown as a square root scale.

The relevant meropenem dosing regimens attaining different PK/PD targets (40%*f*T > MIC, 100%*f*T > MIC, and 100%*f*T > 4 × MIC) were determined by PK model simulation as follows: 75 mg/kg/q6h (40%*f*T > MIC), 75 mg/kg/q2.4h (100%*f*T > MIC), 50 mg/kg/q2.4h (100%*f*T > MIC), and 75 mg/kg/q2h (100%*f*T > 4 × MIC). The simulated concentration–time curves for the above dosing regimens are shown in Figure 2. Of note, the 100%*f*T > MIC target could be achieved by the regimens of 75 mg/kg/q2.4h and 50 mg/kg/q2.4h, and these two regimens were designed to check the dose difference on the efficacy response.

### 2.4. PK/PD Outcomes of Meropenem in Sepsis Rat Model

The observed mean ± standard deviation (SD) concentrations of different treatment groups are shown in Figure 2 and Supplementary Table S1, which is generally in good agreement with the simulated profiles. The individual magnitude of PK/PD index for the treatment groups (75 mg/kg/q6h, 75 mg/kg/q2.4h, 50 mg/kg/q2.4h, and 75 mg/kg/q2h) was determined based on the measured meropenem concentrations and PK modeling, and the calculated target attainments are presented in Table 1. Overall, the actual target attainments of the PK/PD indices for different treatment groups were in good agreement with predefined magnitudes.

**Figure 3:**
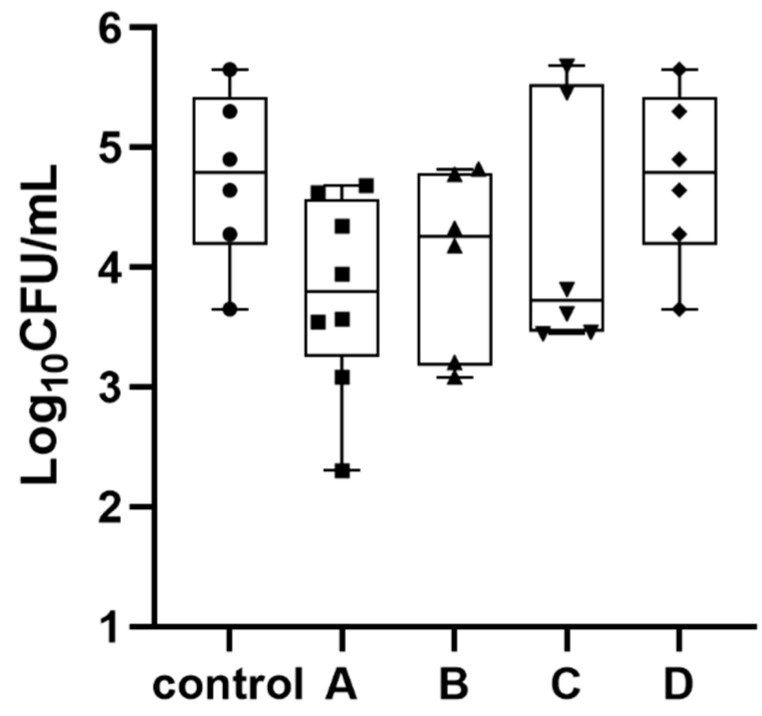
The box-plot distribution of bacteria counts in blood samples from sepsis rats following 12 h of treatment with meropenem. The dosing regimens for the treatment groups of A, B, C, and D were 75 mg/kg/q6h (40%*f*T > MIC), 75 mg/kg/q2.4h (100%*f*T > MIC), 50 mg/kg/q2.4h (100%*f*T > MIC), and 75 mg/kg/q2h (100%*f*T > 4 × MIC), respectively. The control group received equal volume of sterile saline.

The bacterial counts in blood after a 12 h treatment duration are displayed in Figure 3. A large individual difference in bacterial counts was observed in each group. The median bacterial counts in the treatment groups of 75 mg/kg/q6h, 75 mg/kg/q2.4h, and 50 mg/kg/q2.4h were lower than the control group, while similar counts were observed for the control group and 75 mg/kg/q2h treatment group. There were no statistically significant differences between different groups based on a Student’s t-test (*p* > 0.05). No colony was observed on the MHA plates containing 0.375 mg/mL meropenem in any group, indicating no resistance developed during the treatment period.

**Figure 4:**
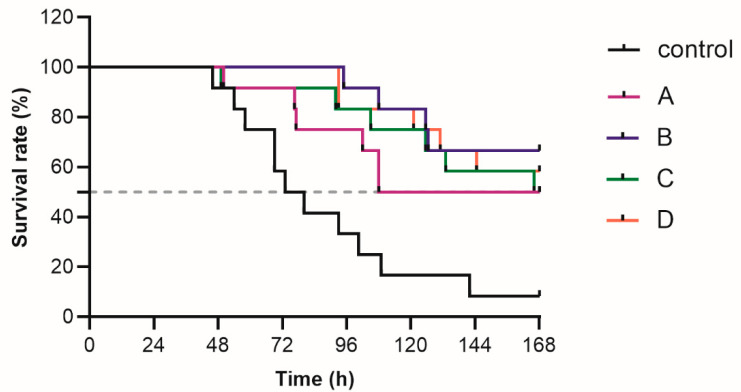
The survival curves of sepsis rats infected with 1 × 10^7^ CFU *Pseudomonas aeruginosa* ATCC9027 and followed with different meropenem regimens attaining various PD targets. A: 75 mg/kg/q6h (40%*f*T > MIC), B: 75 mg/kg/q2.4h (100%*f*T > MIC), C: 50 mg/kg/q2.4h (100%*f*T > MIC), and D: 75 mg/kg/q2h (100%*f*T > 4 × MIC). Control: sterile saline.

The 7-day survival curves of the sepsis rats in different groups are shown in Figure 4. Graphically, the treatment groups showed better survival outcomes than the control group. For instance, the median survival time for the treatment groups of 75 mg/kg/q6h and 50 mg/kg/q2.4h was almost doubled compared to the control group (Table 2). The survival rates of the treatment groups of 75 mg/kg/q2.4h and 75 mg/kg/q2h are all over 50%, so the median survival time was not defined. Among the treatment groups, the groups of 50 mg/kg/q2.4h, 75 mg/kg/q2.4h, and 75 mg/kg/q2h displayed comparable survival outcomes, which were visibly better than that of 75 mg/kg/q6h group. The results of further statistical analysis for the log-rank test and hazard ratio comparison are depicted in Table 2 and Table 3. The log-rank test confirmed that the survival outcomes of the treatment groups versus the control group were indeed statistically significant (Table 2). The hazard ratios of the treatment groups versus the control group were all less than 1, indicating the protective effect of meropenem treatment. The log-rank test showed that survival outcomes between different treatment groups were insignificant (Table 3). However, the hazard ratios of the treatment groups of 75 mg/kg/q2.4h (100%*f*T > MIC) and 75 mg/kg/q2h (100%*f*T > 4 × MIC) versus the 75 mg/kg/q6h group (40%*f*T > MIC) were between 0.51–0.66, demonstrating a potentially beneficial effect of the aggressive PK/PD target attainment.

## 3. Discussion

In the present study, the rat sepsis model was used to evaluate the microbiological and survival outcomes of meropenem dosing regimens attaining different PK/PD targets (40%*f*T > MIC, 100%*f*T > MIC, and 100%*f*T > 4 × MIC). The rat sepsis model was developed to mimic the situations for critically ill patients with severe bacterial infections. The well-known thigh infection model is not considered for the development of a sepsis model because it usually represents local infections [20]. Infections that lead to sepsis most often start in the lung, abdomen, urinary tract, or central nervous system, and the abdomen is the second most common source of sepsis [21]. Currently, there are three main methods for a sepsis animal model, including injection of an exogenous toxin (e.g., lipopolysaccharide), alteration of the endogenous protective barrier (e.g., cecal ligation and puncture, CLP), and infusion or instillation of exogenous bacteria [22]. The CLP-method-induced sepsis is due to polymicrobial infections by translocation of various cecum bacteria into the blood. As we need to design an elegant sepsis model with the infection bacteria presenting a specific MIC (facilitating PK/PD target determination), the intra-abdominal sepsis model by intraperitoneal injection of a single species of bacteria was utilized. *Pseudomonas aeruginosa* was selected as the working bacteria because it is a difficult-to-treat bacterial infection in critically ill sepsis patients and is sensitive to meropenem [23].

In vivo results demonstrated that meropenem treatment significantly improved survival of rats in the sepsis model. No significant differences in survival outcome were observed between different meropenem treatment groups. The sample size was too small to detect a statistical difference in survival outcomes with respect to different PK/PD targets. An increase in sample size would add to the statistical power, but this is limited by the difficulty of the experimental workload. Nevertheless, it is evident that pronounced survival prolongation was observed in the treatment groups of 75 mg/kg/q2.4h (100%*f*T > MIC), 50 mg/kg/q2.4h (100%*f*T > MIC), and 75 mg/kg/q2h (100%*f*T > 4 × MIC) compared to the 75 mg/kg/q6h (40%*f*T > MIC) group, with the time of 75% survival being 105–125.5 h rather than 77 h. From the results of the hazard ratio and the time of 75% survival, the traditional PK/PD target of 40%*f*T > MIC is suboptimal for sepsis patients. It is not surprising that no survival difference was found between the treatment groups of 75 mg/kg/q2.4h (100%*f*T > MIC) and 50 mg/kg/q2.4h (100%*f*T > MIC). As a time-dependent antibiotic, the attained magnitude of the %*f*T > MIC was the same between these two meropenem groups, so the expected bactericidal effect was similar. This indicated that excessive meropenem exposure under the same %*f*T > MIC in sepsis patients could not provide an improved outcome. The comparable survival curves between meropenem groups with PD targets of 100%*f*T > MIC and 100%*f*T > 4 × MIC indicated that the aggressive 100%*f*T > 4 × MIC magnitude could not further improve the survival outcome. However, in vitro studies have demonstrated that 100%*f*T > 4-6 × MIC is needed for preventing the development of resistance of the β-lactams, including carbapenems [18,24]. Using a static-killing set-up, Mouton et al. reported that the regrowing of *Pseudomonas aeruginosa* was observed after a few hours of ceftazidime treatment with a sustained concentration around or slightly above the initial MIC, while a sustained concentration 4 to 5 times the MIC is effective for suppressing the resistance [18]. In an in vitro dynamic killing study, the resistance of a subpopulation of *Pseudomonas aeruginosa* increased after several days of meropenem treatment with a 100%*f*T > 1.7 × MIC target, and the emergence of resistance can be suppressed with a 100%*f*T > 6.2 × MIC target [24]. Although a 100%*f*T > 4 × MIC target provides added value for preventing resistance, the attainment of this target at clinically safe doses is often hard to achieve. In a post hoc analysis of a prospective study, a PD target of 100%*f*T > 4 × MIC is very low for septic patients, even when high-loading meropenem doses are administered over an extended infusion period [25]. In addition, excessive drug exposure might cause adverse effects. In a retrospective study of 130 septic patients treated with meropenem, elevated meropenem trough concentration was associated with increased occurrence of neurological deterioration [26].

The effect of microbiological eradication for different PD targets is inconclusive due to the “irregular” bacterial counts in blood samples. Even in the control group, we failed to observe an obvious growing trend for *Pseudomonas aeruginosa* in the blood in sepsis rats. During the development of the sepsis model, the bacterial counts in blood at several time points (e.g., 2, 4, 8, and 24 h after intraperitoneal injection) were measured, and no clear trends were observed for different inoculum sizes (1 × 10^7^–1 × 10^8^ CFU) of the bacteria. This phenomenon was also observed in other animal studies with *Pseudomonas aeruginosa* as the infection bacteria [27,28], and the intrinsic reason remains unknown. We suspected the following reasons that may partly account for the “irregular” observations: (a) the growth of bacteria in blood is quite complicated and is influenced by many physiological factors, such as immunological situations; (b) the response of the immune system to similar bacterial-burden-induced infection may be quite different in rats, resulting in potential differences in the bactericidal ability of the body system.

Drug resistance was not observed in all treatment groups within the 12 h treatment duration. The selective amplification of the resistance of a subpopulation often occurs after a relative long treatment period, so our results in this rat sepsis model may not reflect the actual resistance situation in sepsis patients because of the short treatment period and the relatively low inoculum size (1 × 10^7^ CFU). To unravel this question, the use of an in vitro hollow-fiber infection model (HFIM) [29], a dynamic killing model that could precisely simulate human PK and readily assess bacterial killing and resistance over long periods (e.g., 14 days), could provide informative data about the microbiological effects of the different PD magnitudes (40%*f*T > MIC, 100%*f*T > MIC, and 100%*f*T > 4 × MIC) of meropenem.

## 4. Materials and Methods

### 4.1. Drug, Organisms, and Media

Meropenem (Aladdin Biochemical Technology Co., Ltd., Shanghai, China) was dissolved in sterile ultrapure water, following serially twofold dilution to the desired concentrations used for in vitro study. For in vivo study, meropenem was reconstituted in sterile 0.9% saline, then it was further diluted to the required concentration according to the milligram-per-kilogram of body weight doses. *Pseudomonas aeruginosa* ATCC 9027 and 27853 (Shanghai Bioresource Collection Center, Shanghai, China) were included in these experiments. The organisms were stored in 40% glycerin at −80 °C prior to use. Microorganisms were incubated, subcultured, and quantified in Mueller-Hinton broth (MHB) and agar (MHA) (Guangdong Huankai Microbial Sci. & Tech. Co., Ltd., Guangdong, China).

### 4.2. Antimicrobial Susceptibility Test

MICs of the aforementioned organisms to meropenem were performed by the agar dilution method according to guidelines of the Clinical and Laboratory Standards Institute (CLSI) [30]. The final concentrations of meropenem ranged from 0.002 to 32 mg/L. Organisms were incubated in MHB at 37 °C to the logarithmic phase. The bacterial suspensions were subsequently diluted with MHB and adjusted to the OD of 0.08–0.13 at a wavelength of 625 nm, resulting in approximately 1 × 10^8^ CFU/mL. From this dilution, 100 μL of the bacterial suspension was further mixed with 900 μL of MHB to yield a 1:10 dilution, and 2 μL of the resulting solution was added to the MHA plate containing serial twofold dilutions of meropenem, resulting in a bacterial density of 1 × 10^4^ CFU/spot. The inoculated plates were then incubated at 37 °C for 16–20 h. In each run, two antimicrobial-free MHA growth control plates were included. MIC was determined as the lowest concentration of meropenem that inhibited visible growth of bacteria. The isolates were tested in triplicate. *Pseudomonas aeruginosa* ATCC 27853 was used as the quality control.

### 4.3. Development of Sepsis Rat Model

Specific-pathogen-free, Sprague–Dawley rats weighting 180–220 g were obtained from Hunan SJA Laboratory animal Co., Ltd. in Changsha, China. All animal experiments and animal care were performed under protocols approved by the Animal Ethics Committee of Central South University. Throughout the experiment, rats were provided with food and water ad libitum.

Rats were transiently rendered neutropenic by intraperitoneal administration of two doses of cyclophosphamide (100 mg/kg on day 0 (5 days before infection) plus 75 mg/kg on day 4 (1 day before infection)) [31]. The glycerol bacteria (*Pseudomonas aeruginosa* ATCC 9027) stored at −80 °C were thawed and then cultured in MHB at 37 °C under a rotation speed of 180 rpm to the logarithmic phase. The fresh broth culture of bacteria was diluted serially with sterile saline to obtain different working suspensions for injection at 1 × 10^8^ CFU/mL, 5 × 10^7^ CFU/mL, 2.5 × 10^7^ CFU/mL, and 1 × 10^7^ CFU/mL. On day 5, the neutropenic rats were randomized into five groups (four treatment groups + one control group), each containing 12 animals (six females and six males). The treatment groups were intraperitoneally injected with 1 mL of *Pseudomonas aeruginosa* ATCC 9027 at different bacterial concentrations, and the control group was intraperitoneally injected with 1 mL sterile saline. After infection, rats were observed for physical conditions and mortality for 7 days. The optimal load of bacterial suspensions for the rat sepsis model was determined based on the criterion of about 50% of lethality rate after 2 to 3 days infection [32].

### 4.4. Pharmacokinetics Study and Calculation of Free Drug Concentration

The pharmacokinetic characteristics of meropenem in the developed rat sepsis model was investigated, and the resulting PK data was subsequently used for compartmental modeling to determine the suitable dosing regimens to attain different PK/PD targets (40%*f*T > MIC, 100%*f*T > MIC, and 100%*f*T > 4 × MIC).

The optimal bacterial load for the sepsis rat model with predefined survival rate determined in section of “2.3 Development of sepsis rat model” was intraperitoneal injection with 1 × 10^7^ CFU of *Pseudomonas aeruginosa* ATCC 9027. For the PK study, six neutropenic rats (male/female, 50/50) were first intraperitoneally injected with 1 × 10^7^ CFU of *Pseudomonas aeruginosa* ATCC 9027. After two hours, the rats were injected subcutaneously with meropenem at a single dose of 100 mg/kg. A small amount of blood (10 μL) was collected through tail vein at 0.083, 0.167, 0.5, 1, 1.5, 2, and 4 h after meropenem administration. Meropenem blood samples were analyzed based on an adapted method of ultra-performance liquid chromatography with photodiode array (UPLC-PDA) for plasma samples [33]. Blood samples (10 μL) were deproteinated with acetonitrile (100 μL) containing 20 μg/mL of metronidazole (internal standard). Chromatographic separation was performed with an ACQUITY UPLC HSS T3 column (2.1 × 50 mm, 1.8 μm) at a flow rate of 0.5 mL/min. The calibration range was 0.3 to 150 μg/mL (r^2^ = 0.999). The intra- and inter-day precisions were less than 9.1%, and the intra- and inter-day accuracies were between 89.0% and 99.3%.

The measured meropenem in blood samples is total concentration. To obtain free meropenem plasma concentration, the protein binding ratio (PPB) and blood-to-plasma concentration ratio (BPR) of meropenem in rats were measured. The conversion of total meropenem blood concentration ($C_{total}$) to free plasma concentration ($C_{free}$) is based on Equation 1:

(1) $$C_{free}=C_{total}/BPR\times \left(1-PPB\right)$$

The protein binding ratio of meropenem in rat plasma was determined by the ultrafiltration method. Briefly, 400 µL of rat plasma containing 10 μg/mL meropenem (*n* = 3) was added onto an Amicon^®^ Ultra 0.5 mL filter (molecular weight cutoff: 30 K, Merck). The sample was incubated at 37 °C and centrifuged at 1500× *g* for 15 min. The filtrate was measured by the UPLC-PDA method.

Blood-to-plasma concentration ratio of meropenem in the rats was measured according to a previous report [34]. In short, 50 μL of meropenem (200 μg/mL) was added to 950 μL of fresh rat blood, and then the blood was incubated at 37 °C for 10 min under a rotating speed of 500 rpm (AS ONE^®^ Block Bath Shaker MyBL-100CS, Shanghai, Japan). Afterwards, the blood sample was centrifuged, and the obtained plasma was analyzed by the UPLC-PDA method.

### 4.5. Pharmacokinetic Modeling and Dosing Regimen Simulation

Compartmental modeling was performed for the measured meropenem blood concentration data using Phoenix WinNonlin software (Version 8.1, Pharsight Corporation, Princeton, USA). The mean values of the relevant PK parameters were calculated.

By combining the determined MIC of *Pseudomonas aeruginosa* ATCC 9027 (0.125 mg/L), PPB (15%), and BPR (0.65) of meropenem, the relevant meropenem dosing regimens attaining different PK/PD targets (40%*f*T > MIC, 100%*f*T > MIC, and 100%*f*T > 4 × MIC) were determined by PK model simulation using R Version 4.2.0 (R Foundation for Statistical Computing, Vienna, Austria).

### 4.6. Microbiological and Survival Outcomes of Meropenem in Sepsis Rat Model

Sixty neutropenic rats were randomized into five groups (four treatment groups + one control group), each containing 12 animals (male/female, 50/50). The neutropenic rats were intraperitoneally injected with 1 × 10^7^ CFU of *Pseudomonas aeruginosa* ATCC 9027. After two hours, the four treatment groups were given different meropenem regimens (described in Section 3 Pharmacokinetic Parameters of Meropenem and Dosing Regimen Determination) via subcutaneous administration for a period of 12 h, and the control animals were injected with sterile saline.

After the injection, a small amount of blood (10 μL) at several time points (0.083, 0.5, and 1.5 h after the first dose and 0.167, 1, and 2 h after the last dose) was collected from the tail vein of meropenem-dosed animals. These blood samples were measured to verify whether the targeted PK/PD indices were achieved in each treatment group. At 12 h after the treatment, 100 μL of blood was collected from the tail vein of each animal for the quantification of viable organisms. The samples were diluted 10-fold with sterile saline, and 100 μL of the dilutions was spread onto the MHA plates (in triplicate) and cultured at 37 °C for 24 h for viable organism counts. The remainder of the diluted samples (100 μL each in triplicate) was inoculated on MHA plates that contained meropenem at a concentration of 0.375 mg/mL (3 × MIC of *Pseudomonas aeruginosa* ATCC 9027) and cultured for 72 h for resistance estimation.

The mortality of the animals was monitored for 7 days, and the times of death were carefully monitored. A Kaplan–Meier (KM) survival curve for each group was constructed using GraphPad Prism v.8.1 (GraphPad Software, San Diego, CA, USA). The log-rank test was conducted to test difference in survival between different groups, and a *p* value <0.05 was considered statistically significant. Also, the hazard ratio was calculated between different groups.

## 5. Conclusions

In an intra-abdominal sepsis rat model, we demonstrated that an inferior survival outcome was observed for rats attaining the traditional 40%*f*T > MIC target compared with the aggressive PD targets of 100%*f*T > MIC and 100%*f*T > 4 × MIC, while the 100%*f*T > 4 × MIC target does not provide further improvement of survival compared with the target of 100%*f*T > MIC.

## Figures and Tables

**Table 1:** The actual target attainment of different PK/PD targets in sepsis rats receiving various meropenem dosing regimens.

Subject	40%*f*T > MIC (75 mg/kg/q6h)	100%*f*T > MIC (75 mg/kg/q2.4h)	100%*f*T > MIC (50 mg/kg/q2.4h)	100%*f*T > 4 × MIC (75 mg/kg/q2h)
1	41.0%	100%	100%	100%
2	78.1%	100%	100%	100%
3	60.7%	100%	80.6%	100%
4	49.7%	100%	86.8%	94.4%
5	46.9%	100%	87.1%	100%
6	45.8%	100%	100%	100%
7	56.1%	92.9%	100%	100%
8	58.1%	100%	100%	100%
9	42.3%	100%	100%	100%
10	39.1%	95.5%	100%	93.7%
11	43.8%	100%	98.6%	100%
12	64.8%	100%	100%	89.6%

**Table 2:** Statistical results of survival outcomes between meropenem treatment groups and the control in sepsis rat model.

	Control	40%*f*T > MIC (75 mg/kg/q6h)	100%*f*T > MIC (75 mg/kg/q2.4h)	100%*f*T > MIC (50 mg/kg/q2.4h)	100%*f*T > 4 × MIC (75 mg/kg/q2h)
Log-rank test (*p* value)		0.0187	0.0004	0.0051	0.0008
Median survival time (h)	76.5	138	Undefined	167	Undefined
Hazard ratio (95% CI)		0.3306 (0.1240, 0.8814)	0.1709 (0.0578, 0.5059)	0.2779 (0.1017, 0.7594)	0.2096 (0.0730, 0.6017)

CI: confidence interval.

**Table 3:** The statistical results of survival outcomes between different meropenem treatment groups in sepsis rat model.

	B/A	C/A	D/A	B/C	B/D	D/C
Log-rank test (*p* value)	0.2831	0.8144	0.4819	0.4234	0.7042	0.6704
Hazard ratio (95% CI)	0.5132 (0.1470, 1.7920)	0.8742 (0.2812, 2.7180)	0.6587 (0.2004, 2.1650)	0.6016 (0.1739, 2.0820)	0.7768 (0.2103, 2.8700)	0.7744 (0.2373, 2.5270)

CI: confidence interval. A = 40%*f*T > MIC (75 mg/kg/q6h), B = 100%*f*T > MIC (75 mg/kg/q2.4h), C = 100%*f*T > MIC (50 mg/kg/q2.4h), D = 100%*f*T > 4 × MIC (75 mg/kg/q2h).

## Data Availability

The data used to support the findings of this study are available from the corresponding author upon request.

## Supplementary Materials

The following are available online at https://www.mdpi.com/article/10.3390/antibiotics11111660/s1, Table S1: The measured concentrations (mean ± SD) of meropenem in sepsis rats treated with four dosing regimens attaining different PK/PD targets.
