# Supplementary material for: Defining Exposure Predictors of Meropenem That Are Associated with Improved Survival for Severe Bacterial Infection: A Preclinical PK/PD Study in Sepsis Rat Model
Source: Antibiotics (Basel). 2022 Nov 19;11(11):1660. doi: 10.3390/antibiotics11111660 (PMC9686672; doi:10.3390/antibiotics11111660)
Supplement: Supplementary file 1 [file antibiotics-11-01660-s001.zip › antibiotics-1981638-supplementary.pdf]

**Table S1.** The measured concentrations (mean±SD) of meropenem in sepsis rats treated with four dosing regimens attaining different PK/PD targets.

| **PK/PD targets** | | | | | | | |
|---|---|---|---|---|---|---|---|
| 40%fT>MIC (75 mg/kg q6h) | | 100%fT>MIC (50 mg/kg q2.4h) | | 100%fT>MIC (75 mg/kg q2.4h) | | 100%fT>4×MIC (75 mg/kg q2h) | |
| Time (h) | Concentration （mg/L） | Time (h) | Concentration （mg/L） | Time (h) | Concentration （mg/L） | Time (h) | Concentration （mg/L） |
| 0.083 | 7.92±1.49 | 0.083 | 7.18±4.09 | 0.083 | 19.2±3.48 | 0.083 | 11.83±4.74 |
| 0.5 | 12.88±0.70 | 0.5 | 7.67±2.04 | 0.5 | 12.44±4.56 | 0.5 | 12.35±2.15 |
| 1.5 | 2.22±0.70 | 1.5 | 1.52±0.76 | 1 | 5.56±2.32 | 1.5 | 1.58±0.48 |
| 6.167 | 17.15±4.14 | 9.767 | 14.29±5.32 | 9.683 | 10.59±4.65 | 10.167 | 22.66±11.12 |
| 7 | 6.52±1.50 | 10.6 | 4.36±1.50 | 10.6 | 6.78±3.20 | 11 | 6.47±1.28 |
| 8 | 1.52±0.72 | 11.6 | 0.58±0.15 | 11.6 | 2.19±1.45 | 11.917 | 1.59±0.93 |

*Each treatment group has 12 rats, while at each time-point six rats were sampled for concentration measurement.
